# Ethyl 6-amino-5-cyano-2-methyl-4-propyl-4*H*-pyran-3-carboxyl­ate

**DOI:** 10.1107/S1600536809043748

**Published:** 2009-10-28

**Authors:** Qun-Di Yu, Ke-Xin Li, Yun-Yu Liu

**Affiliations:** a Food Science and Pharmacy College, Zhejiang Ocean University, Zhoushan 316000, People’s Republic of China; b People’s Hospital of Jilin Province, Changchun 130021, People’s Republic of China; c Department of Chemistry, Northeast Normal University, Changchun 130024, People’s Republic of China

## Abstract

The pyran ring of the title compound, C_13_H_18_N_2_O_3_, is almost planar (r.m.s. deviation = 0.059 Å). The crystal packing is stabilized by N—H⋯O and N—H⋯N hydrogen bonds.

## Related literature

Ethyl 6-amino-5-cyano-2-methyl-4-propyl-4*H*-pyran-3-carb­ox­yl­ate and its derivatives are widely utilized as organic inter­mediates, see: Liang *et al.* (2009 ▶).
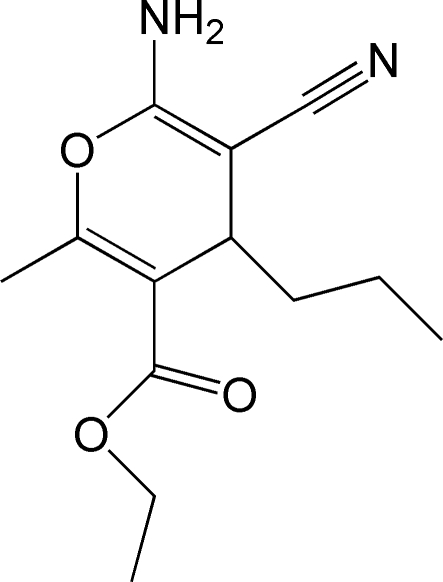


## Experimental

### 

#### Crystal data

C_13_H_18_N_2_O_3_
*M* *_r_* = 250.15
Triclinic, 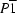

*a* = 8.1172 (9) Å
*b* = 8.7956 (9) Å
*c* = 11.2877 (19) Å
α = 106.082 (12)°
β = 107.274 (12)°
γ = 103.315 (9)°
*V* = 695.20 (19) Å^3^
*Z* = 2
Mo *K*α radiation
μ = 0.09 mm^−1^
*T* = 293 K
0.25 × 0.23 × 0.20 mm

#### Data collection

Bruker SMART APEX CCD area-detector diffractometer
Absorption correction: multi-scan (*SADABS*; Bruker, 1998 ▶) *T*_min_ = 0.65, *T*_max_ = 0.87
5049 measured reflections
2826 independent reflections
1577 reflections with *I* > 2σ(*I*)
*R*_int_ = 0.023

#### Refinement

*R*[*F* ^2^ > 2σ(*F* ^2^)] = 0.046
*wR*(*F* ^2^) = 0.129
*S* = 0.89
2826 reflections
172 parameters
H atoms treated by a mixture of independent and constrained refinement
Δρ_max_ = 0.13 e Å^−3^
Δρ_min_ = −0.22 e Å^−3^

### 

Data collection: *SMART* (Bruker, 1998 ▶); cell refinement: *SAINT* (Bruker, 1998 ▶); data reduction: *SAINT*; program(s) used to solve structure: *SHELXS97* (Sheldrick, 2008 ▶); program(s) used to refine structure: *SHELXL97* (Sheldrick, 2008 ▶); molecular graphics: *SHELXTL* (Sheldrick, 2008 ▶); software used to prepare material for publication: *SHELXTL*.

## Supplementary Material

Crystal structure: contains datablocks global, I. DOI: 10.1107/S1600536809043748/bt5109sup1.cif

Structure factors: contains datablocks I. DOI: 10.1107/S1600536809043748/bt5109Isup2.hkl

Additional supplementary materials:  crystallographic information; 3D view; checkCIF report

## Figures and Tables

**Table 1:** Hydrogen-bond geometry (Å, °)

*D*—H⋯*A*	*D*—H	H⋯*A*	*D*⋯*A*	*D*—H⋯*A*
N2—H2*A*⋯O1^i^	0.805 (18)	2.088 (19)	2.881 (2)	168.2 (17)
N2—H2*B*⋯N1^ii^	0.85 (2)	2.21 (2)	3.035 (3)	164.4 (17)

Symmetry codes: (i) 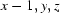; (ii) 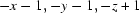.

**Fig. 1.**
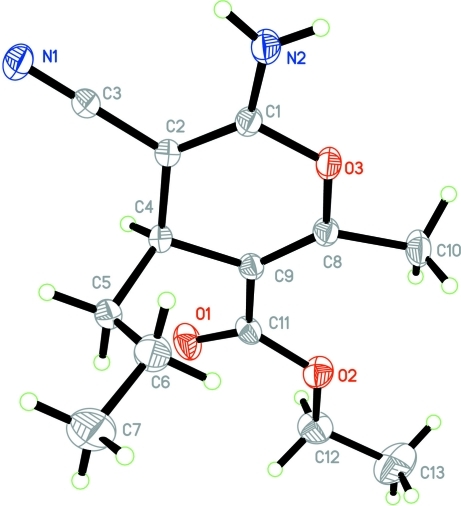
The structure of the title compound, showing the atomic numbering scheme. Displacement ellipsoids are drawn at the 30% probability level.

## supplementary crystallographic information

### Comment

Ethyl 6-amino-5-cyano-2-methyl-4-propyl-4H-pyran-3-carboxylate and its derivatives are widely utilized as organic intermediates (Liang *et al.*, 2009).

The pyran ring of the title compound, C_13_H_18_N_2_O_3_, is almost planar (r.m.s. deviation 0.059Å). The crystal packing is stabilized by N-H···O and N-H···N hydrogen bonds.

### Experimental

A mixture of butyraldehyde (1.0 mmol), malononitrile (1.0 mmol) and acetyl acetate (1.0 mmol) was dissolved in 5 mL dimethylformamide and catalytic amount of piperidine (0.2 mmol) was added at room temperature under stirring. After 2h, the reaction mixture was poured into water and extracted with CH_2_Cl_2_. The combined organic phase was washed with water, dried over MgSO_4_, filtered and concentrated in vacuo. The crude product was purified by silica column chromatography. Yield: 87%. Pure product was dissolved in a mixture of petroleum ether. The single-crystals were obtained by slow evaporation of the solvents.

### Refinement

All H atoms on C atoms were positioned geometrically (C—H = 0.93-0.98Å) and refined as riding, with *U*_iso_(H)=1.2*U*_eq_(C) or *U*_iso_(H)=1.5*U*_eq_(C_methyl_). The H atoms bonded to N were freely refined.

### Crystal data

**Table:**

C_13_H_18_N_2_O_3_	*Z* = 2
*M**_r_* = 250.15	*F*(000) = 268
Triclinic, *P*1	*D*_x_ = 1.195 Mg m^−^^3^
Hall symbol: -P 1	Mo *K*α radiation, λ = 0.71073 Å
*a* = 8.1172 (9) Å	Cell parameters from 2826 reflections
*b* = 8.7956 (9) Å	θ = 3.0–26.4°
*c* = 11.2877 (19) Å	µ = 0.09 mm^−^^1^
α = 106.082 (12)°	*T* = 293 K
β = 107.274 (12)°	Block, colorless
γ = 103.315 (9)°	0.25 × 0.23 × 0.20 mm
*V* = 695.20 (19) Å^3^	

### Data collection

**Table:**

Bruker APEX CCD area-detector diffractometer	2826 independent reflections
Radiation source: fine-focus sealed tube	1577 reflections with *I* > 2σ(*I*)
graphite	*R*_int_ = 0.023
φ and ω scans	θ_max_ = 26.4°, θ_min_ = 2.6°
Absorption correction: multi-scan (*SADABS*; Bruker, 1998)	*h* = −10→10
*T*_min_ = 0.65, *T*_max_ = 0.87	*k* = −10→10
5049 measured reflections	*l* = −13→14

### Refinement

**Table:**

Refinement on *F*^2^	Primary atom site location: structure-invariant direct methods
Least-squares matrix: full	Secondary atom site location: difference Fourier map
*R*[*F*^2^ > 2σ(*F*^2^)] = 0.046	Hydrogen site location: inferred from neighbouring sites
*wR*(*F*^2^) = 0.129	H atoms treated by a mixture of independent and constrained refinement
*S* = 0.89	*w* = 1/[σ^2^(*F*_o_^2^) + (0.0757*P*)^2^] where *P* = (*F*_o_^2^ + 2*F*_c_^2^)/3
2826 reflections	(Δ/σ)_max_ < 0.001
172 parameters	Δρ_max_ = 0.13 e Å^−^^3^
0 restraints	Δρ_min_ = −0.21 e Å^−^^3^

### Special details

**Table:**

Geometry. All esds (except the esd in the dihedral angle between two l.s. planes) are estimated using the full covariance matrix. The cell esds are taken into account individually in the estimation of esds in distances, angles and torsion angles; correlations between esds in cell parameters are only used when they are defined by crystal symmetry. An approximate (isotropic) treatment of cell esds is used for estimating esds involving l.s. planes.
Refinement. Refinement of F^2^ against ALL reflections. The weighted R-factor wR and goodness of fit S are based on F^2^, conventional R-factors R are based on F, with F set to zero for negative F^2^. The threshold expression of F^2^ > 2sigma(F^2^) is used only for calculating R-factors(gt) etc. and is not relevant to the choice of reflections for refinement. R-factors based on F^2^ are statistically about twice as large as those based on F, and R- factors based on ALL data will be even larger.

### Fractional atomic coordinates and isotropic or equivalent isotropic displacement parameters (Å^2^)

**Table:**

	*x*	*y*	*z*	*U*_iso_*/*U*_eq_	
C1	−0.2429 (2)	−0.09571 (19)	0.63342 (17)	0.0444 (4)	
C2	−0.1346 (2)	−0.18666 (19)	0.61071 (16)	0.0428 (4)	
C3	−0.2165 (2)	−0.3640 (2)	0.55184 (18)	0.0507 (5)	
C4	0.0703 (2)	−0.10825 (19)	0.65363 (16)	0.0416 (4)	
H4	0.1012	−0.1530	0.5765	0.050*	
C5	0.1778 (2)	−0.1557 (2)	0.76684 (17)	0.0513 (5)	
H5A	0.1418	−0.2774	0.7362	0.062*	
H5B	0.3075	−0.1117	0.7850	0.062*	
C6	0.1500 (3)	−0.0922 (3)	0.8948 (2)	0.0716 (6)	
H6A	0.0195	−0.1271	0.8756	0.086*	
H6B	0.1976	0.0298	0.9305	0.086*	
C7	0.2423 (4)	−0.1545 (3)	1.0004 (2)	0.1038 (9)	
H7A	0.2186	−0.1102	1.0791	0.156*	
H7B	0.3723	−0.1175	1.0223	0.156*	
H7C	0.1945	−0.2753	0.9666	0.156*	
C8	0.0021 (2)	0.1608 (2)	0.70345 (17)	0.0446 (4)	
C9	0.1207 (2)	0.08115 (19)	0.69043 (16)	0.0404 (4)	
C10	0.0223 (3)	0.3431 (2)	0.7354 (2)	0.0670 (6)	
H10A	−0.0868	0.3524	0.6792	0.101*	
H10B	0.1258	0.3989	0.7199	0.101*	
H10C	0.0409	0.3947	0.8274	0.101*	
C11	0.3091 (2)	0.1666 (2)	0.70545 (18)	0.0466 (4)	
C12	0.5617 (3)	0.4184 (3)	0.7863 (2)	0.0737 (6)	
H12A	0.5646	0.4016	0.6984	0.088*	
H12B	0.6491	0.3749	0.8326	0.088*	
C13	0.6091 (4)	0.5982 (3)	0.8618 (3)	0.1117 (9)	
H13A	0.7300	0.6584	0.8713	0.168*	
H13B	0.6061	0.6135	0.9486	0.168*	
H13C	0.5222	0.6403	0.8149	0.168*	
N2	−0.4192 (2)	−0.1503 (2)	0.61418 (18)	0.0606 (5)	
N1	−0.2822 (2)	−0.5075 (2)	0.50412 (19)	0.0763 (6)	
O3	−0.17565 (15)	0.07590 (13)	0.68500 (12)	0.0530 (3)	
O1	0.39733 (18)	0.09129 (16)	0.65942 (14)	0.0694 (4)	
O2	0.37809 (16)	0.33123 (15)	0.77399 (14)	0.0612 (4)	
H2A	−0.472 (2)	−0.083 (2)	0.6160 (17)	0.055 (5)*	
H2B	−0.485 (3)	−0.253 (3)	0.585 (2)	0.062 (6)*	

### Atomic displacement parameters (Å^2^)

**Table:**

	*U*^11^	*U*^22^	*U*^33^	*U*^12^	*U*^13^	*U*^23^
C1	0.0435 (10)	0.0357 (9)	0.0503 (11)	0.0118 (8)	0.0178 (8)	0.0123 (8)
C2	0.0427 (10)	0.0356 (9)	0.0466 (10)	0.0149 (8)	0.0133 (8)	0.0131 (8)
C3	0.0410 (10)	0.0451 (11)	0.0606 (12)	0.0174 (9)	0.0140 (9)	0.0157 (9)
C4	0.0439 (10)	0.0366 (9)	0.0449 (10)	0.0182 (8)	0.0168 (8)	0.0129 (7)
C5	0.0490 (11)	0.0426 (10)	0.0593 (12)	0.0179 (8)	0.0138 (9)	0.0205 (9)
C6	0.0846 (16)	0.0705 (14)	0.0584 (13)	0.0299 (12)	0.0213 (11)	0.0261 (11)
C7	0.130 (2)	0.109 (2)	0.0662 (15)	0.0379 (18)	0.0184 (15)	0.0482 (16)
C8	0.0421 (10)	0.0380 (9)	0.0533 (11)	0.0135 (8)	0.0195 (8)	0.0152 (8)
C9	0.0412 (10)	0.0380 (9)	0.0441 (10)	0.0155 (8)	0.0161 (8)	0.0173 (8)
C10	0.0593 (13)	0.0393 (10)	0.1052 (17)	0.0212 (9)	0.0362 (12)	0.0222 (11)
C11	0.0453 (10)	0.0486 (11)	0.0552 (11)	0.0215 (9)	0.0197 (9)	0.0281 (9)
C12	0.0484 (12)	0.0710 (14)	0.0989 (17)	0.0079 (11)	0.0285 (12)	0.0374 (13)
C13	0.0887 (19)	0.0724 (17)	0.134 (3)	−0.0179 (14)	0.0439 (18)	0.0169 (17)
N2	0.0453 (10)	0.0414 (10)	0.0917 (13)	0.0153 (9)	0.0289 (9)	0.0167 (9)
N1	0.0583 (11)	0.0403 (10)	0.1089 (15)	0.0124 (9)	0.0212 (10)	0.0126 (10)
O3	0.0443 (7)	0.0348 (6)	0.0765 (9)	0.0148 (6)	0.0265 (6)	0.0115 (6)
O1	0.0578 (9)	0.0646 (9)	0.1057 (12)	0.0336 (7)	0.0447 (8)	0.0355 (8)
O2	0.0470 (7)	0.0474 (8)	0.0829 (10)	0.0073 (6)	0.0285 (7)	0.0184 (7)

### Geometric parameters (Å, °)

**Table:**

C1—N2	1.329 (2)	C8—C9	1.333 (2)
C1—C2	1.350 (2)	C8—O3	1.3862 (19)
C1—O3	1.3638 (19)	C8—C10	1.501 (2)
C2—C3	1.415 (2)	C9—C11	1.476 (2)
C2—C4	1.512 (2)	C10—H10A	0.9600
C3—N1	1.145 (2)	C10—H10B	0.9600
C4—C9	1.522 (2)	C10—H10C	0.9600
C4—C5	1.540 (2)	C11—O1	1.2122 (18)
C4—H4	0.9800	C11—O2	1.326 (2)
C5—C6	1.502 (3)	C12—O2	1.456 (2)
C5—H5A	0.9700	C12—C13	1.467 (3)
C5—H5B	0.9700	C12—H12A	0.9700
C6—C7	1.517 (3)	C12—H12B	0.9700
C6—H6A	0.9700	C13—H13A	0.9600
C6—H6B	0.9700	C13—H13B	0.9600
C7—H7A	0.9600	C13—H13C	0.9600
C7—H7B	0.9600	N2—H2A	0.805 (18)
C7—H7C	0.9600	N2—H2B	0.85 (2)
			
N2—C1—C2	128.59 (16)	C9—C8—C10	130.81 (16)
N2—C1—O3	110.12 (13)	O3—C8—C10	107.27 (12)
C2—C1—O3	121.28 (15)	C8—C9—C11	123.41 (14)
C1—C2—C3	117.85 (15)	C8—C9—C4	122.47 (15)
C1—C2—C4	122.94 (14)	C11—C9—C4	114.08 (12)
C3—C2—C4	119.09 (12)	C8—C10—H10A	109.5
N1—C3—C2	179.8 (2)	C8—C10—H10B	109.5
C2—C4—C9	109.31 (11)	H10A—C10—H10B	109.5
C2—C4—C5	111.67 (13)	C8—C10—H10C	109.5
C9—C4—C5	112.78 (13)	H10A—C10—H10C	109.5
C2—C4—H4	107.6	H10B—C10—H10C	109.5
C9—C4—H4	107.6	O1—C11—O2	121.58 (16)
C5—C4—H4	107.6	O1—C11—C9	122.36 (16)
C6—C5—C4	114.61 (13)	O2—C11—C9	116.06 (13)
C6—C5—H5A	108.6	O2—C12—C13	107.97 (17)
C4—C5—H5A	108.6	O2—C12—H12A	110.1
C6—C5—H5B	108.6	C13—C12—H12A	110.1
C4—C5—H5B	108.6	O2—C12—H12B	110.1
H5A—C5—H5B	107.6	C13—C12—H12B	110.1
C5—C6—C7	113.67 (18)	H12A—C12—H12B	108.4
C5—C6—H6A	108.8	C12—C13—H13A	109.5
C7—C6—H6A	108.8	C12—C13—H13B	109.5
C5—C6—H6B	108.8	H13A—C13—H13B	109.5
C7—C6—H6B	108.8	C12—C13—H13C	109.5
H6A—C6—H6B	107.7	H13A—C13—H13C	109.5
C6—C7—H7A	109.5	H13B—C13—H13C	109.5
C6—C7—H7B	109.5	C1—N2—H2A	117.1 (13)
H7A—C7—H7B	109.5	C1—N2—H2B	124.7 (13)
C6—C7—H7C	109.5	H2A—N2—H2B	117.0 (18)
H7A—C7—H7C	109.5	C1—O3—C8	120.17 (11)
H7B—C7—H7C	109.5	C11—O2—C12	116.81 (13)
C9—C8—O3	121.91 (14)		

### Hydrogen-bond geometry (Å, °)

**Table:**

*D*—H···*A*	*D*—H	H···*A*	*D*···*A*	*D*—H···*A*
N2—H2A···O1^i^	0.805 (18)	2.088 (19)	2.881 (2)	168.2 (17)
N2—H2B···N1^ii^	0.85 (2)	2.21 (2)	3.035 (3)	164.4 (17)

Symmetry codes: (i) *x*−1, *y*, *z*; (ii) −*x*−1, −*y*−1, −*z*+1.
